# Seasonal and Regional Dynamics of the Intestinal Microbiota in *Schizothorax nukiangensis* from the Nujiang River

**DOI:** 10.3390/ani15070961

**Published:** 2025-03-27

**Authors:** Fengyue Zhu, Jie Ma, Mingyang Xue, Weitong Xu, Wenzhi Liu, Yong Zhou, Mingdian Liu, Yuding Fan

**Affiliations:** 1 Yangtze River Fisheries Research Institute, Chinese Academy of Fishery Sciences, Wuhan 430223, China; zhufy@yfi.ac.cn (F.Z.); majie2673@stu.ouc.edu.cn (J.M.); xmy@yfi.ac.cn (M.X.); xuweitong@yfi.ac.cn (W.X.); liuwenzhialisa@yfi.ac.cn (W.L.); zhouy@yfi.ac.cn (Y.Z.); 2 Key Laboratory of Tropical Aquatic Germplasm of Hainan Province, Sanya Oceanographic Institution, Ocean University of China, Sanya 572024, China

**Keywords:** *Schizothorax nukiangensis*, Nujiang River, metagenomics, intestinal microbiota, seasonal and regional dynamics

## Abstract

The dynamic alterations in intestinal microbiota can provide insights into the adaptive relationships between these microorganisms and their hosts in response to environmental changes. *Schizothorax nukiangensis* is widely distributed throughout the Nujiang River and exhibits numerous unique adaptations. In this study, we collected samples of *S. nukiangensis* across different seasons and regions within the Nujiang River to comprehensively elucidate the diversity and composition of its intestinal microbiota using metagenomic technology. The results indicated that Firmicutes and Proteobacteria predominated at the phylum level, while *Priestia*, *Bacillus*, and *Aeromonas* were the most abundant genera identified. Notably, the relative abundance of these microorganisms varied significantly across different seasons and regions. From autumn through spring and into summer, the predominant microorganisms shifted from Firmicutes to Proteobacteria. Biomarker analysis revealed that Firmicutes (including the class bacilli and the genera *Priestia* and *Bacillus*) exhibited a higher relative abundance within the upstream group, where categories related to amino acid metabolism and carbohydrate metabolism were significantly enriched. Conversely, Proteobacteria (including several potential pathogens, such as *Saezia*, *Pantoea*, *Lelliotia*, and *Aeromonas* genera) showed an increased relative abundance within downstream groups, where disease-related categories exhibited significant enrichment. Our findings significantly enhance our understanding of how *S. nukiangensis* adapts to its environment, providing valuable data support for the conservation of *S. nukiangensis* and for ecological security assessment of the Nujiang River.

## Simple Summary

*Schizothorax nukiangensis* is widely distributed throughout the Nujiang River and exhibits numerous unique adaptations. To understand its adaptation, we comprehensively elucidated the diversity and composition of its intestinal microbiota using metagenomic technology. The results indicated that the intestinal microbiota of *S. nukiangensis* was predominantly composed of Firmicutes and Proteobacteria at the phylum level. From autumn to spring and summer, there was a shift in predominant microorganisms from Firmicutes to Proteobacteria. Furthermore, Firmicutes (including the class bacilli—specifically, the genera *Priestia* and *Bacillus*) exhibited a higher relative abundance in the upstream group. In contrast, Proteobacteria (which includes several potential pathogens, such as *Saezia*, *Pantoea*, *Lelliotia*, and *Aeromonas*) demonstrated increased relative abundance within downstream groups. Our findings enhance the understanding of how *S. nukiangensis* adapts to its environment.

## 1. Introduction

The intestinal microbiota of organisms represents one of the most influential symbiotic communities, playing a crucial role in host health by regulating metabolism, energy utilization and storage, nutrient absorption, immune function, and disease prevention [1,2]. The gut microbiota of fish can be significantly influenced by various factors, including host genotype, immunity, diet, ecotype, and abiotic environmental conditions [3,4,5,6,7]. Animals are particularly susceptible to fluctuations in food resources and environmental temperatures. Wildlife often encounters temporal variations in food availability and frequently adjusts their diets accordingly [6]. A previous study indicated that alterations in the intestinal microbiota of wildlife may serve as an adaptive mechanism for securing adequate nutrition during seasonal fluctuations related to changes in food availability [8]. Furthermore, the intestinal microbiota of animals responds indirectly to environmental temperatures while being directly affected by the physiological responses of hosts to seasonal shifts in food sources [9]. Seasonal variations in host diets may also induce changes in metabolic pathways, leading to functional and compositional differences within the intestinal microbiota [10]. Consequently, dynamic changes within the intestinal microbiota can provide insights into the adaptive relationship between these microbial communities and their hosts as they respond to environmental changes.

The Nujiang River is an important international waterway that traverses the Tibet Autonomous Region and Yunnan Province in China. Upon entering Myanmar, it is referred to as the Salween River, ultimately discharging into the Andaman Sea within the Indian Ocean. The topography of the Nujiang River basin exhibits higher elevations in the northwest and lower altitudes in the southeast, while its climate is characterized by complexity and variability influenced by both terrain features and atmospheric circulation patterns. The unique and diverse habitats found within this basin provide crucial environments for the growth and reproduction of various endemic fish species. Reports indicate that there are a total of 85 indigenous fish species inhabiting this river in China, many of which are endemic [11]. Due to its rich ecological resources, this area has been designated as one of the world’s biodiversity hotspots [12]. However, recent years have seen a dramatic decline in fish populations within the Nujiang River due to factors such as overfishing, mining activities, hydropower station construction on certain tributaries, and environmental disruption [13]. In order to scientifically manage and protect the fish resources in the Nujiang River basin, researchers have conducted extensive studies on the diversity and distribution patterns of fish over many years [11,14,15]. Research indicates that the Nujiang River can be divided at Lushui City. The upstream section in Tibet, located above Lushui, lies within the heart of the Qinghai–Tibet Plateau and is characterized by a cold climate with prolonged periods of ice and snow. The upstream Yunnan section above Lushui features high mountains and deep valleys with rapid water flow and pronounced vertical climatic variations. The fish species inhabiting these two sections are predominantly cold water species typical of the Qinghai–Tibet Plateau. In contrast, the downstream section below Lushui has a lower elevation but presents a complex terrain influenced by the southwest monsoon, resulting in hot and rainy conditions. Consequently, fish species found here exhibit distinct characteristics associated with South Asian subtropical riverine ecosystems. The significant longitudinal environmental gradient within this basin impedes exchanges among fish communities throughout the Nujiang River basin [11,15]. Notably, *Schizothorax nukiangensis*, a cold water fish endemic to the Nujiang River with high economic value, is widely distributed across both Tibet and Yunnan regions, exhibiting many unique adaptations [16]. Therefore, *S. nukiangensis* serves as an exemplary representative for studying and evaluating ecological security within the Nujiang River ecosystem. Previous studies on *S. nukiangensis* mainly focused on genetics [16], biology [17,18], plateau adaptation [19], etc. However, there remains a notable gap regarding studies focused on intestinal microbiota that limits our understanding of how *S. nukiangensis* adapts to its environment.

Therefore, in this study, we selected *S. nukiangensis* as the research subject and employed metagenomic sequencing technology to investigate its intestinal microbiota. We analyzed the variations in intestinal microbial composition and function across different seasons and river segments. Our findings will enhance our understanding of how *S. nukiangensis* adapts to its environment, providing valuable data support for the conservation of this species, as well as for ecological security assessments of the Nujiang River.

## 2. Materials and Methods

### 2.1. Sample Collection

**Figure 1:**
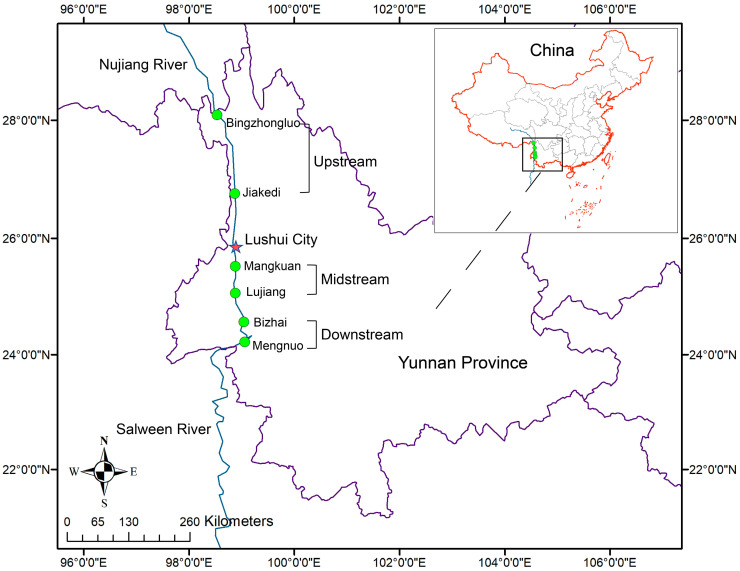
Sampling locations along the upstream, midstream, and downstream reaches of the Yunnan section of the Nujiang River. The sampling sites are marked by green dots and labeled with the names of nearby towns.

We collected samples of *S. nukiangensis* inhabiting the Nujiang River in Yunnan Province during October 2023, April 2024, and August 2024, representing consecutive autumn, spring, and summer seasons, respectively. Healthy fish (weight = 20~90 g) were obtained from six sampling sites along the Nujiang River. These six sites were categorized into three regions based on their geographical locations: upstream, midstream, and downstream (Figure 1). In each region, four individual *S. nukiangensis* samples were collected during every season, except for the downstream region during the summer. This is because *S. nukiangensis* cannot be captured in the downstream region during the summer. Following anesthesia with a solution of 100 mg/L tricaine methanesulfonate (MS-222; Sigma, St. Louis, MO, USA), samples of entire contents of the intestine were collected from these fish using sterile scissors and forceps. The intestinal contents were then immediately transferred to enzyme-free 1.5 mL EP tubes and subsequently frozen in liquid nitrogen. All tubes were stored at −80 °C until DNA extraction was performed. During the fish sampling process, field observations recorded data on the water temperature and altitude. Detailed sampling information is provided in Table S1.

### 2.2. DNA Extraction

Total genomic DNA was extracted from the intestinal contents using the PowerSoil DNA Isolation Kit (MO BIO, Carlsbad, CA, USA) in accordance with the manufacturer’s instructions. The extracted DNA was assessed utilizing 1.5% agarose gels, a NanoPhotometer spectrophotometer (IMPLEN, Westlake Village, CA, USA), and the Qubit dsDNA Assay Kit on a Qubit 2.0 fluorometer (Life Technologies, Carlsbad, CA, USA). Subsequently, the DNA samples were stored at −80 °C until further analysis.

### 2.3. Metagenome Sequencing, Data Preprocessing, and Assembly

DNA samples derived from the intestinal contents were subsequently subjected to metagenomic sequencing. A total of 1 µg of DNA per sample was utilized as the input material for sequencing library construction, employing the NEBNext Ultra™ DNA Library Prep Kit (New England Biolabs, Ipswich, MA, USA). The DNA samples were fragmented to an average size of 350 bp through sonication. Following fragmentation, end repair was performed using an end repair mix, and indexing adapters were ligated to the ends of the DNA fragments. After a quality assessment, the prepared libraries were sequenced on an Illumina NovaSeq platform (Illumina, San Diego, CA, USA), generating paired-end raw reads. To obtain valid data for subsequent analysis, data preprocessing was conducted using FastUniq (version 1.1) software to eliminate PCR duplicates and Trimmomatic (version 0.39) for adapter removal and quality filtering. Post-filtering, KneadData (version 0.10.0) was employed to remove contaminating sequences originating from human and host sources in order to yield clean data. Subsequently, these clean data were assembled into contigs utilizing MEGAHIT software (version 1.2.9). The N50 length metric was applied to assess the assembly quality. Contigs with lengths ≥ 500 bp were used for Open Reading Frame (ORF) prediction via Prodigal (version 2.6.3). Unigenes intended for further annotation were generated using CD-HIT (version 4.8.1), while Salmon (version 1.10.1) facilitated the calculation of Transcripts Per Million (TPM) values for Unigenes across each individual sample.

### 2.4. Metagenomic and Statistical Analysis

For composition annotation, Unigenes were subjected to BLAST (version 2.2.31+) analysis against sequences from Bacteria, Eukaryota, Archaea, and viruses extracted from the NR database of the NCBI. This was accomplished using DIAMOND software (version 2.0.11) in conjunction with the lowest common ancestor (LCA) algorithm. For functional analysis, DIAMOND was employed to annotate Unigenes based on the Kyoto Encyclopedia of Genes and Genomes (KEGG), with the parameters set to an E-value threshold of ≤1 × 10^−5^. Subsequently, the best Blast Hit was utilized for further examination of the blast results. Sequences that lack annotation at this level in the database, or whose corresponding species have not been definitively classified at this level, are categorized as unclassified. The abundance of each taxon group is obtained by summing up the abundances of the genes belonging to that taxon group. The relative abundance at different functional hierarchies was calculated as the sum of the relative abundances of the genes annotated at each specific functional level. Biomarker species between groups were performed via LEfSe (version 1.1.11), with a Linear Discriminant Analysis (LDA) score threshold established at ≥4. The unpaired *t*-test was used to assess the differences between the two groups.

## 3. Results

### 3.1. Overview of Metagenome Sequencing Results and Microbial Diversity

From the metagenomic sequencing of 32 intestinal microbial samples, a total of 281 GB of raw data were generated. Following quality control measures, removal of host sequences, and assembly processes, we obtained 45,569,932 contigs (Table S2). Subsequently, we calculated the alpha diversity for all samples (Table S3). Good’s coverage values for all samples exceeded 0.99, indicating that sufficient sequencing coverage was achieved and that the results accurately reflect the true composition of the microbiota in these samples.

**Figure 2:**
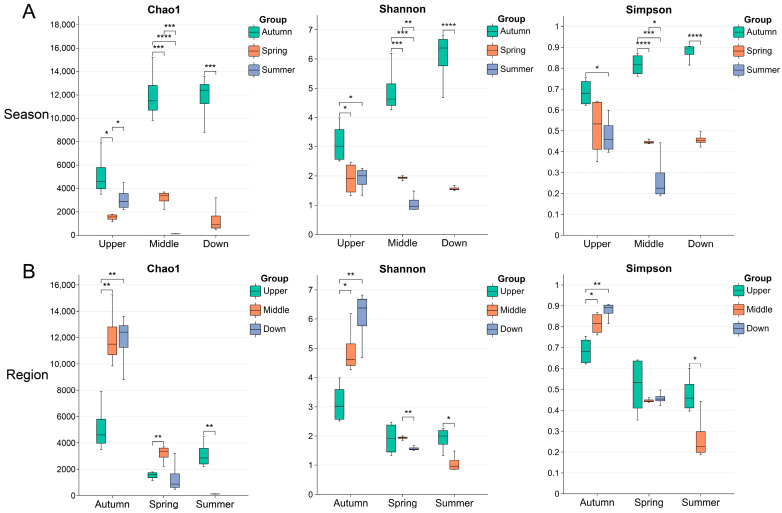
Alpha diversity of intestinal microbiota of *S. nukiangensis* in different seasons (**A**) and regions (**B**). The unpaired *t*-test was used to assess the differences between the two groups: * *p* < 0.05; ** *p* < 0.01; *** *p* < 0.001; **** *p* < 0.0001.

We performed a significance analysis on microbial diversity indices, including Chao1, Shannon, and Simpson, across various seasons and regions. The results indicated significant differences in microbial diversity among all the samples influenced by seasonal and regional factors. In terms of seasonal variations, microbial diversity demonstrated a decreasing trend in the following order: autumn > spring > summer (Figure 2A). Regarding regional differences, during autumn, downstream sites exhibited higher diversity compared to upstream sites; however, this difference diminished in the spring. Additionally, the midstream sites showed lower diversity than the upstream sites during the summer (Figure 2B).

### 3.2. Composition of the Intestinal Microbiota in S. nukiangensis

**Figure 3:**
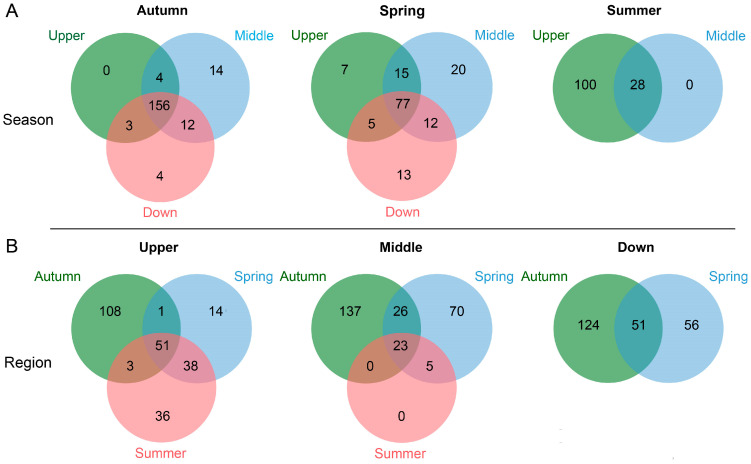
Venn diagrams illustrating the seasonal overlap of intestinal microbiota in *S. nukiangensis* (**A**) and regions (**B**) at the phylum level.

In the analysis of 32 samples, a total of 12 kingdoms, 300 phyla, 224 classes, 460 orders, 1104 families, 5140 genera, and 50,551 species were identified. Among the samples collected during the same season, most phyla classifications of the intestinal microbiota were consistently observed across the three regions (Figure 3A). However, when examining samples from the same region across different seasons, only a limited number of phyla classifications were found to be common (Figure 3B). These findings suggest that seasonal variations exert a greater influence on the intestinal microbiota of *S. nukiangensis* compared to regional factors.

**Figure 4:**
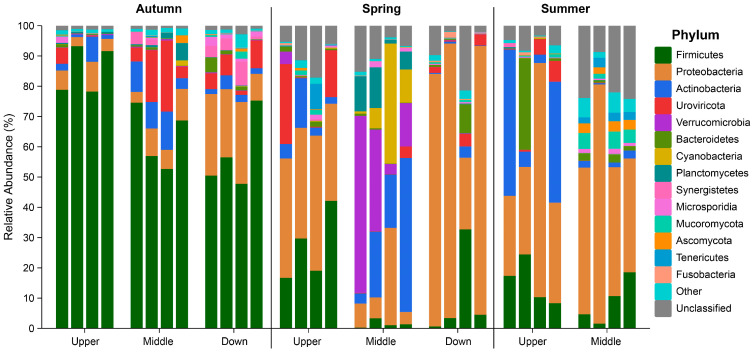
Intestinal microbiota composition of *S. nukiangensis* at the phylum level.

At the phylum level across all samples, the intestinal microbiota was predominantly composed of members from the Firmicutes and Proteobacteria phyla. Minor contributions were observed from Actinobacteria, Verrucomicrobia, Bacteroidetes, Cyanobacteria, and Planctomycetes within the bacterial super kingdom, as well as Uroviricota from the viral super kingdom; however, variations in composition were observed among individual samples collected during different seasons and from various regions (Figure 4 and Table 1). In the autumn samples, a similar microbial composition was noted across all regions, with Firmicutes being the dominant phylum. Conversely, in the spring samples, Proteobacteria emerged as the predominant phylum within the intestinal microbiota. The Uroviricota phylum, primarily consisting of bacterial viruses (phages), was identified in the upstream samples. The midstream samples exhibited the highest microbial diversity, with the dominant phyla including Actinobacteria, Verrucomicrobia, Cyanobacteria, and Planctomycetes. During the summer sampling period, both Proteobacteria and Firmicutes remained prevalent alongside Actinobacteria. One noteworthy observation could be made: Firmicutes represented the largest proportion within the autumn groups, whereas Proteobacteria predominated during the spring and summer (Table 1).

**Figure 5:**
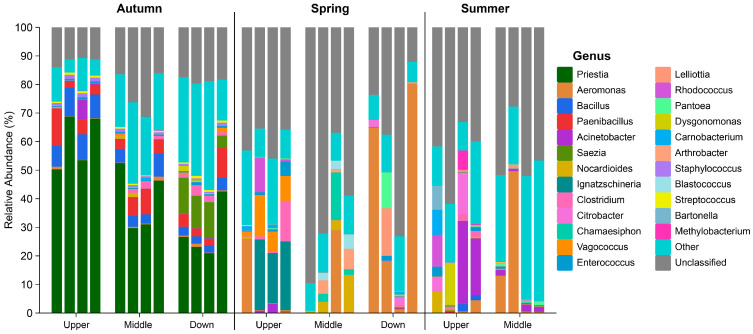
Intestinal microbiota composition of *S. nukiangensis* at the genus level.

At the genus level, significant variations were observed among the major genera across different seasonal groups (Figure 5). The autumn group was predominantly characterized by the genera *Priestia*, *Bacillus*, *Paenibacillus*, and *Clostridium*, which played a major role in metabolism. Notably, the downstream samples contained the potential pathogen *Saezia*. In contrast, within the spring group, *Aeromonas* emerged as a common bacterium across various regions. Furthermore, the upstream samples revealed the presence of another potential pathogen, *Ignatzschineria*. The midstream samples were primarily dominated by *Arthrobacter* from the Actinobacteria phylum and *Chamaesiphon* from the Cyanobacteria phylum; additionally, potential pathogens *Lelliottia* and *Pantoea* were prevalent in the downstream samples. During the summer months, the midstream samples exhibited a dominance of *Aeromonas* (predominantly, *Aeromonas hydrophila*), while *Acinetobacter*, *Citrobacter*, and *Bartonella* prevailed in the upstream samples.

At the species level, the relative abundance of *Priestia megaterium* in the autumn group reached 33%, while those of other species did not exceed 5% in any group (Table S4).

### 3.3. Analysis of Biomarker Species in Intestinal Microbiota

**Figure 6:**
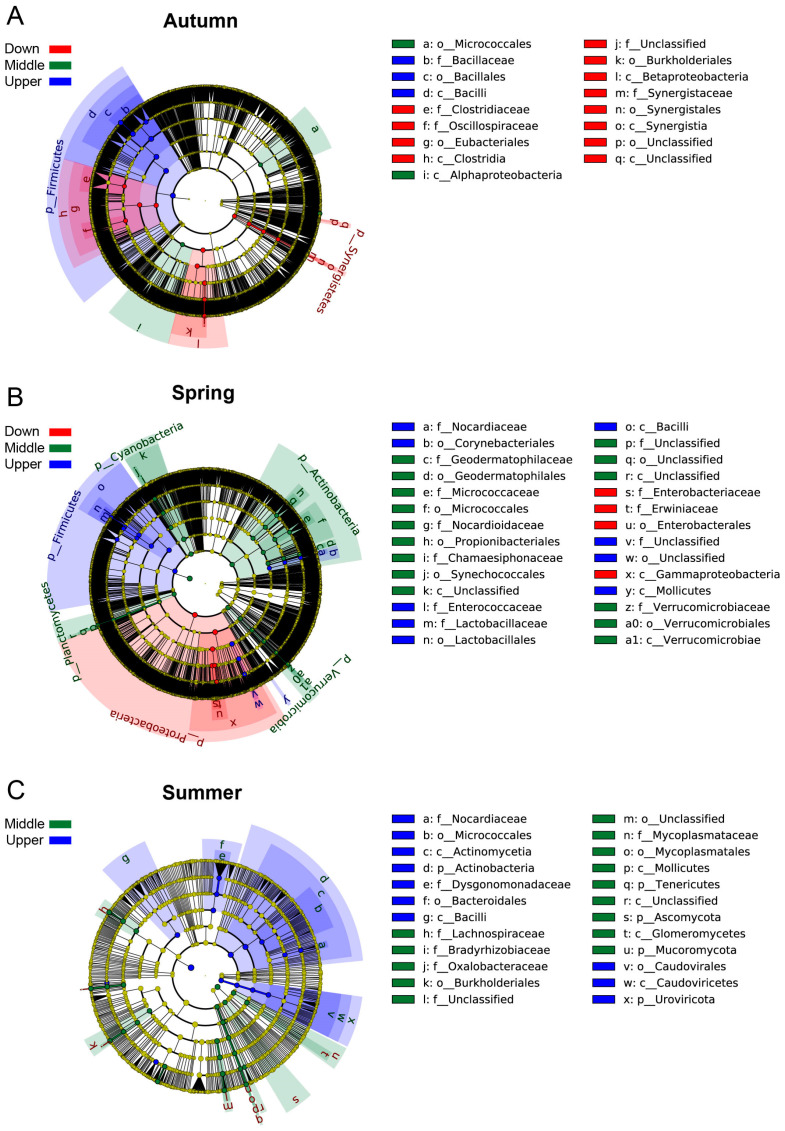
LEfSe analysis in the autumn (**A**), spring (**B**), and summer (**C**) groups of the *S. nukiangensis* intestinal microbiota. The concentric circles radiating outward represent taxonomic hierarchies from phylum to genus. Red nodes indicate significantly different microbial taxa in the downstream group, green nodes denote those in the midstream group, and blue nodes represent significantly different microbial taxa in the upstream group. The criteria for significant differences were defined as a (1) LDA score ≥ 4 and (2) non-parametric Kruskal–Wallis rank sum test showing significant abundance differences (*p* < 0.05) relative to any other group.

LEfSe analysis was conducted on the intestinal microbial metagenomic sequencing data of *S. nukiangensis* to identify microbial species exhibiting significant differences across various seasonal and regional groups. The findings revealed that, during the autumn, the Bacilli class exhibited the highest relative abundance in the upstream group, while *Aeromonas* phage species were most abundant in the midstream group. In contrast, the *Sazea* genus, Burkholderiales order, and Synergistia class demonstrated peak relative abundance within the downstream group (Figure 6A). In the spring, both the Firmicutes phylum and *Ignatzschineria* genus showed the highest relative abundance in the upstream group. The midstream group was characterized by elevated levels of Actinobacteria, Verrucomicrobia, Cyanobacteria, and Planctomycetes phyla. Meanwhile, Proteobacteria phylum encompassed potential pathogens such as *Pantoea*, along with *Lelliottia* genus and *Erwiniaceae* family, in the downstream group (Figure 6B). During the summer months, Actinobacteria and Uroviricota phyla, alongside Bacilli class and Bacteroidales order, had their highest relative abundances recorded in the upstream group; conversely, Mucoromycota and Ascomycota fungi, as well as Tenericutes phylum together with Burkholderiales order, reached their maximum relative abundances within the midstream group (Figure 6C). Detailed information on significantly different species is shown in Table S5.

### 3.4. Functional Analysis of the Intestinal Microbiota in S. nukiangensis

**Figure 7:**
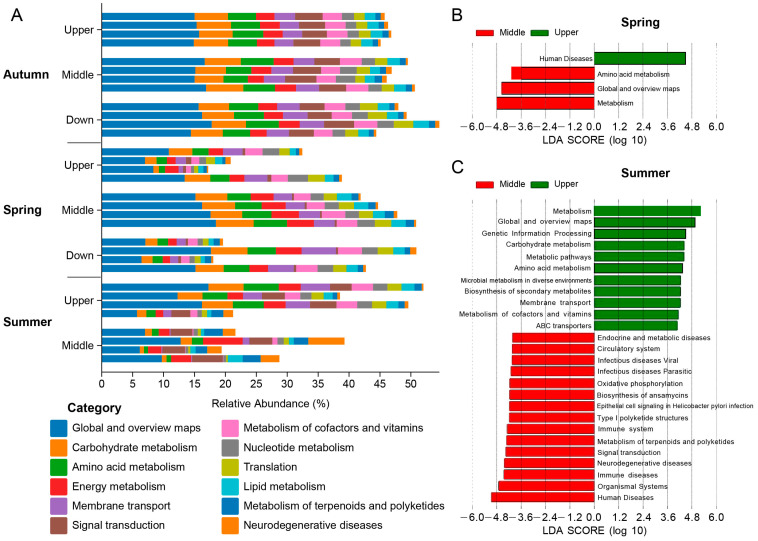
KEGG functional analysis of the intestinal microbiota of *S. nukiangensis*. (**A**) KEGG functional analysis based on microbial abundance. (**B**,**C**) KEGG categories specific to the spring and summer, respectively, as identified through LEfSe analysis based on the microbial abundance across different regional groups.

The KEGG functional analysis indicated that the intestinal microbial functions of *S. nukiangensis* primarily centered on metabolic processes (Figure 7A). In the spring, both metabolism and amino acid metabolism categories were significantly enriched in the midstream group, whereas the human disease category was notably enriched upstream (Figure 7B). During the summer, categories associated with diseases (e.g., viral infectious diseases, parasitic infectious diseases, and immune disorders) were enriched in the midstream group, while those related to metabolism (e.g., carbohydrate metabolism, amino acid metabolism, and coenzyme and vitamin metabolism) were predominant upstream (Figure 7C).

## 4. Discussion

Intestinal microbiota exerts a significant influence on the host’s metabolism and immune system, with the formation of their community structure being a complex process shaped by genetic, dietary, and environmental factors. Consequently, the dynamic changes in intestinal microbiota can serve as indicators to infer the adaptive relationships between these microorganisms and their hosts in response to environmental fluctuations. While numerous studies have extensively investigated the intestinal microbial structures of aquatic animals, this research represents the first examination of wild fish inhabiting the highly variable habitat environment of the Nujiang River. In this study, we collected samples from *S. nukiangensis*, a representative species of this river, across different seasons and regions. We comprehensively analyzed its intestinal microbiota diversity and composition using metagenomic technology. The results revealed that, at the phylum level, Proteobacteria and Firmicutes predominated within the intestinal microbiota of *S. nukiangensis*; at the genus level, *Priestia*, *Bacillus*, and *Aeromonas* were the most prevalent. Notably, there were significant variations in the relative abundance of these microorganisms across different seasons and regions. The seasonal and regional dynamics observed in the intestinal microbiota provide valuable insights into how *S. nukiangensis* adapts to its environment.

### 4.1. Seasonal Dynamics of the Intestinal Microbiota in S. nukiangensis

Seasonal variations in the intestinal microbiota of wild aquatic animals, such as crucian carp [20], tench [21], and Chinese mitten crab [22], have been documented in numerous studies. However, these seasonal differences are not entirely uniform across species. This variability may be attributed to fish adjusting their activity patterns, physiological requirements, feeding behaviors, and ecological habits in response to seasonal changes [23]. In our study, the composition of the intestinal microbiota of *S. nukiangensis* during autumn exhibited significant differences compared to those observed in spring and summer. Notably, the alpha diversity was higher in autumn than in both spring and summer. Firmicutes predominated within the autumn group, whereas Proteobacteria were more prevalent in the spring and summer groups. Additionally, there was an increase in the proportion of Actinobacteria, Cyanobacteria, Verrucomicrobia, and Planctomycetes within the spring group. These variations may be linked to seasonal environmental changes and food availability. *S. nukiangensis* primarily feeds on aquatic insect larvae, benthic invertebrates, and attached algae; they also scrape off attached algae and organic debris from stones at the riverbed [18]. This feeding behavior enables them to thrive in rivers like the Nujiang River that are clear but possess relatively limited food resources. However, it is noteworthy that, during spring (February to April) and summer (July to August), high rainfall occurs within the Yunnan section of the Nujiang River basin, while autumn experiences lower precipitation levels [24]. Such conditions may hinder predation by *S. nukiangensis*, as their food sources could shift towards plant litter or animal detritus, along with organic debris transported by erosion. However, in the spring and summer, the water flow caused by rain constantly erodes the soil on the shore, bringing a large amount of sediment into the water, resulting in increased turbidity [25]. At this time, the food sources for *S. nukiangensis* may shift to include plant and animal litter, as well as organic debris transported by water flow. This change may not favor the predation of *S. nukiangensis*. Our sampling also corroborated this observation; specifically, the feeding intensity of *S. nukiangensis* in autumn was found to be higher than that observed in spring and summer (Table S1).

Previous studies have demonstrated that starvation generally leads to a decrease in gut microbial diversity and affects the structure of the microbiota. Firmicutes are primarily responsible for the hydrolysis of proteins and carbohydrates [26], while Proteobacteria utilize alternative energy sources to provide additional energy for the host during periods of starvation [27]. Mekuchi et al. [28] investigated the microbiota and host metabolism in leopard coral grouper. It was found that Proteobacteria dominated during fasting, whereas Firmicutes were more prevalent during feeding. Furthermore, the microbial diversity under feeding conditions is greater than that observed during fasting, a phenomenon also noted in grass carp [29], grouper [30], and Atlantic salmon [31]. The reduced yet varied food sources available in the spring and summer may further complicate the intestinal microbiota composition of *S. nukiangensis*. Actinobacteria are widely distributed across various habitats, such as soil, aquatic environments, plant litter, compost, and food; they can enter hosts through ingestion and have been identified as the dominant intestinal microorganisms in many fish species, including rainbow trout and largemouth bass [32,33]. The high abundance of Cyanobacteria may be attributed to their elevated presence in water environments during the spring and summer; a similar phenomenon has been observed in Asian carp [34]. Previous research has indicated that cellulase secreted by gamma-proteobacteria (particularly from the genus *Aeromonas*) plays a crucial role in assisting hosts with breaking down plant cell walls, as well as decomposing ingested cellulose and hemicellulose [35,36]. Dietary changes resulting from seasonal variations have also been documented in other plateau *schizothorax* species [37], which may reflect an environmental adaptation strategy among these fish.

### 4.2. Regional Dynamics of the Intestinal Microbiota in S. nukiangensis

Habitat is a significant factor influencing the composition of the gut microbiota, as evidenced by numerous studies [38,39]. In our research, we observed considerable environmental variation across different regions. The upstream region is characterized by high mountains and deep valleys with rapid water flow, whereas the downstream region features lower elevations with complex terrain and a hot, rainy climate [11,15]. During our field observations, we also noted that the water temperature at the downstream location is approximately 3 °C higher than that at the upstream location (Table S1). Additionally, human activities have a notable impact on water quality. The midstream and downstream areas are influenced by the surrounding sand processing operations and animal husbandry practices, resulting in elevated concentrations of ammonia nitrogen and silicate [25]. Our findings indicate that the genera *Saezia*, *Pantoea*, and *Lelliotia*; the family *Erwiniaceae*; and the order Burkholderiales exhibited the highest relative abundance in either the midstream or downstream groups. Bacteria belonging to the Burkholderiales order (including the *Saezia* genus) are commonly found in soil and aquatic environments and encompass various pathogenic species [40,41]. The *Erwiniaceae* family, along with *Pantoea* and *Lelliotia* genera, belong to the Enterobacterales order, known for being widespread opportunistic pathogens affecting plants, animals, and humans [42,43,44]. Previous studies have demonstrated that *Aeromonas* and *Pantoea* genera correlate positively with the nitrogen concentration levels while being enriched in the downstream section of the Nujiang River [25]. Furthermore, approximately 80% of gut microbes were shared with microbial communities present in aquatic environments [7]. Consequently, the intestinal infections observed in *S. nukiangensis* may be attributed to the accumulation of pathogens present in midstream and downstream waters. However, the pathogenicity of these bacteria towards *S. nukiangensis* remains uncertain and necessitates further verification through single-strain culture infection experiments.

Throughout three seasons of observation, we consistently noted that the Firmicutes (especially the class bacilli) proportions were higher in the upstream groups compared to those from the midstream or downstream locations. Firmicutes play a dominant role among carnivorous, omnivorous, and herbivorous animals; however, their proportion diminishes sequentially from herbivores to omnivores to carnivores [45]. In the upper reaches of the Yunnan section of the Nujiang River, there is a significant elevation drop accompanied by rapid water flow, which may pose challenges for *S. nukiangensis* in terms of predation. Consequently, the most readily available food source for *S. nukiangensis* appears to be algae that grow on the surfaces of immobile rocks. The genera *Priestia* and *Bacillus* within the Firmicutes phylum facilitate the hydrolysis of proteins and carbohydrates in their hosts [26]. *S. nukiangensis* utilizes these Firmicutes microorganisms in its intestines to aid in food digestion and energy supplementation, enabling it to withstand cold, complex, and harsh external environments. In turn, these Firmicutes microorganisms rely on the energy and nutrients derived from their host’s food intake to ensure their own survival and evolution. This mutualistic relationship may explain why Firmicutes are predominant among upstream populations.

In contrast, Actinobacteria, Cyanobacteria, Verrucomicrobia, Planctomycetes, Mucoromycota, and Ascomycota—members of the super kingdom Fungi— are enriched in midstream groups, indicating a diverse array of food sources available in this region. The diversity observed within the midstream communities suggests that habitat characteristics here serve as a transitional zone between those found upstream and downstream.

Furthermore, we did not capture any *S. nukiangensis* specimens downstream during the summer months. Based on our observations regarding intestinal fullness among captured fish, as well as evidence suggesting an increase in potential pathogens within intestinal microbiota from spring to summer among the midstream groups, we reasonably speculate that the survival conditions for *S. nukiangensis* downstream during the summer may be alarmingly poor.

## 5. Conclusions

This study constitutes the first comprehensive investigation into the seasonal and regional dynamics of the intestinal microbiota in *S. nukiangensis* from the Nujiang River. The results indicated that the intestinal microbiota of *S. nukiangensis* was predominantly composed of Firmicutes and Proteobacteria at the phylum level, with *Priestia*, *Bacillus*, and *Aeromonas* identified as the most abundant genera. Notably, from autumn to spring and summer, there was a shift in predominant microorganisms from Firmicutes to Proteobacteria. Furthermore, Firmicutes (including the class bacilli—specifically, the genera *Priestia* and *Bacillus*) exhibited a higher relative abundance in the upstream group. In contrast, Proteobacteria (which includes several potential pathogens, such as *Saezia*, *Pantoea*, *Lelliotia*, and *Aeromonas*) demonstrated increased relative abundance within the downstream groups. These findings provide comprehensive insights into the dynamics of intestinal microbiota in *S. nukiangensis* and enrich our understanding of fish biology under extreme natural geography and climate conditions in the Nujiang River from a novel perspective. Our research enhances the understanding of how *S. nukiangensis* adapts to its environment while also providing critical data support for conservation efforts regarding *S. nukiangensis*, as well as ecological security assessments for the Nujiang River.

## Figures and Tables

**Table 1:** Microbial composition at the phylum level, exhibiting an average relative abundance exceeding 1% across all samples.

Phylum	Relative Abundance (%)			
	Autumn	Spring	Summer	Average
Firmicutes	68.7 ± 15.7 ^A^	12.9 ± 14.8 ^B^	12.0 ± 7.6 ^B^	33.6
Proteobacteria	11.5 ± 8.9 ^B^	40.9 ± 31.1 ^A^	46.7 ± 20.7 ^A^	31.3
Actinobacteria	4.8 ± 4.0	10.4 ± 14.8	12.9 ± 19.5	8.9
Uroviricota	6.4 ± 7.2	4.7 ± 8.1	1.6 ± 2.7	4.6
Verrucomicrobia	0.2 ± 0.1	9.5 ± 18.3	0.0 ± 0.0	3.7
Bacteroidetes	0.2 ± 0.5	4.9 ± 11.5	0.1 ± 0.1	2.0
Cyanobacteria	0.9 ± 1.6	2.8 ± 4.9	0.1 ± 0.1	2.0
Planctomycetes	0.9 ± 1.3	1.3 ± 2.6	4.8 ± 10.3	1.4
Sum	93.7 ± 5.0	87.4 ± 8.9	78.2 ± 16.7	87.5

Different capital letters indicate extremely significant differences (*p* < 0.01) within the same taxa across different seasons.

## Data Availability

Raw metagenomic sequence data are available at the NCBI under BioProject accession PRJNA1202519.

## Supplementary Materials

The following supporting information can be downloaded at https://www.mdpi.com/article/10.3390/ani15070961/s1: Table S1: Detailed information on the sampling of intestinal samples from different regions in Nujiang River during different seasons; Table S2: Detailed information of metagenomic sequencing on all intestinal samples from different regions in Nujiang River during different seasons.; Table S3. Detailed information of alpha diversity on all intestinal samples from different regions in Nujiang River during different seasons; Table S4: Information of top10 species with the highest relative abundance in different season; Table S5: Biomarker species identified by LEfSe from different regions in Nujiang River during different seasons.
